# Metagenome Assembly and Metagenome-Assembled Genome Sequences from a Historical Oil Field Located in Wietze, Germany

**DOI:** 10.1128/MRA.00333-20

**Published:** 2020-05-21

**Authors:** Michael O. Eze, Stephan A. Lütgert, Hannes Neubauer, Angeliki Balouri, Alina A. Kraft, Anja Sieven, Rolf Daniel, Bernd Wemheuer

**Affiliations:** a Genomic and Applied Microbiology, Georg-August University of Göttingen, Göttingen, Germany; b Göttingen Genomics Laboratory, Georg-August University of Göttingen, Göttingen, Germany; c Department of Earth and Environmental Sciences, Macquarie University, Sydney, NSW, Australia; d German Oil Museum, Wietze, Germany; Loyola University Chicago

## Abstract

Crude oil-polluted sites are a global threat, raising the demand for remediation worldwide. Here, we investigated a crude oil metagenome from a former borehole in Wietze, Germany, and reconstructed 42 metagenome-assembled genomes, many of which contained genes involved in crude oil degradation with a high potential for bioremediation purposes.

## ANNOUNCEMENT

Bioremediation of crude oil-contaminated sites is highly investigated due to severe pollution levels in various ecosystems worldwide. It can be enhanced by the application of microorganisms, and thus it is important to discover novel microbes capable of crude oil degradation (1).

Three crude oil-contaminated samples were taken on 11 October 2016 from a former borehole (52.6592N, 9.8323E) located at a historical oil field in Wietze, Germany (https://www.erdoelmuseum.de). Approximately 5 g of contaminated soil was taken per sample, transported to the laboratory on ice, and stored at −20°C. Environmental DNA was extracted from 100 mg of soil using the PowerSoil DNA extraction kit as recommended by the manufacturer (Qiagen, Hilden, Germany). Paired-end sequencing libraries were constructed using the Nextera DNA sample preparation kit (Illumina, San Diego, CA, USA) and the following Nextera DNA indices: N708/N508 (sample 1), N709/N508 (sample 2), and N710/N508 (sample 3). Paired-end sequencing was performed using a HiSeq 2500 instrument (rapid run mode, 500 cycles), as recommended by the manufacturer (Illumina), and resulted in 46,673,322 paired-end reads (sample 1, 16,094,584 reads; sample 2, 17,883,658 reads; sample 3, 12,695,080 reads). Reads were processed with Trimmomatic version 0.36 (2). Processing included the removal of adapter sequences and low-quality regions. Default parameters were used for all software unless otherwise specified. The quality of the processing was confirmed using FastQC version 0.91. A total of 42,049,950 paired-end reads and 1,147,707 unpaired reads were retained and assembled using metaSPAdes version 3.13.2 (3). Assembly resulted in 1,544,944 scaffolds; of these, 22,257 were larger than 2,500 bp. Coverage information for each scaffold was determined using Bowtie 2 version 2.3.2 (4) and SAMtools version 1.7 (5). The average sequencing depth was approximately 7×. Metagenome-assembled genomes (MAGs) were reconstructed with MetaBAT version 2.12.1 (6). MAG quality was determined using CheckM version 1.0.13 (7). Only MAGs with a completeness minus contamination of more than 50% and a contamination rate of less than 7% were considered for further analysis. MAGs were classified taxonomically using GTDB-Tk version 1.0.2 and the Genome Taxonomy Database (GTDB) (release 86) (8, 9), resulting in 6 archaeal MAGs and 36 bacterial MAGs. Archaeal MAGs were classified as members of the *Euryarchaeota* (1 MAG), *Halobacterota* (3 MAGs), and *Thermoplasmatota* (2 MAGs). Bacterial MAGs belonged to *Actinobacteriota* (4 MAGs), *Bacteroidota* (5 MAGs), *Chloroflexota* (5 MAGs), *Desulfobacterota* (4 MAGs), *Firmicutes* (2 MAGs), *Omnitrophota* (1 MAG), *Patescibacteria* (1 MAG), *Proteobacteria* (10 MAGs), *Spirochaetota* (1 MAG), *Synergistota* (1 MAG), and *Thermotogota* (1 MAG). One bacterial MAG was assigned to an unclassified taxon associated with *Nitrospirae*. After annotation with Prodigal version 2.6.3 (10), functional annotation was performed with DIAMOND version 0.9.29 (11) and the KEGG database (October 2018 release) (12). Functional analysis revealed that all MAGs obtained possess genes involved in xenobiotic degradation. One MAG assigned to *Rugosibacter*, a genus of known xenobiotic degraders (13), showed the highest abundance of pathways associated with xenobiotic degradation (11.8%).

### Data availability.

Raw sequencing data are available at the NCBI Sequence Read Archive (SRA) under accession numbers SRR10568503, SRR10568510, and SRR10568511. The metagenome assembly and the MAGs are available at GenBank under accession numbers WOYI00000000 and WOYJ00000000 to WOZY00000000, respectively. Further genome characteristics and the functional annotation are publicly available at the Göttingen Research Online Database (https://doi.org/10.25625/VX8836).
